# Research and design of TiN/TiAlN coated integral cemented carbide reamers under high cutting-speed and high feed-rate machining conditions

**DOI:** 10.1038/s41598-025-16424-4

**Published:** 2025-08-21

**Authors:** ChaoKuan Zhu, Ju Huang, Yong Zhang

**Affiliations:** Institute of Mechanical Engineering, Chongqing Technology and Business University, 19 Xuefu Avenue, Nan’an District, Chongqing, 400067 China

**Keywords:** Integral cemented carbide reamer, TiN/TiAlN coated, High cutting-speed, High feed-rate, Mechanical engineering, Materials science

## Abstract

The development of high-performance reamers made of Cemented carbide and coating materials is an important direction in mechanical finishing. Due to the complex structure of reamers, it is difficult to manufacture reamers with good performance in practice. This paper takes the high cutting- speed and high feed-rate mechanical finishing of 45 steel as the experimental condition and the production site machining as the background to scientifically investigate the factors affecting the performance of the TiN/TiAlN coated Integral Cemented carbide reamer. Firstly, the TiN/TiAlN coated cemented carbide reamer was designed and manufactured. During the experimental processing, shrinkage hole over-tolerance problems occurred rapidly. By analyzing the cutting characteristics of the reamer, the mechanical model of the cutting edge of the reamer was established, and the principle of chipping was analyzed. The results were consistent with the magnified fracture morphology of the cutting edge. In response to the accelerated wear caused by poor cooling and lubrication, an internal cooling reamer structure was adopted, and the fluid mechanics model of the Integral reamer was constructed. The important structural parameters were determined, and the importance of adequate cooling and lubrication was clarified. By clarifying the factors affecting the processing performance, the machining capacity of this type of reamer was maximized.

## Introduction

**Fig. 1:**
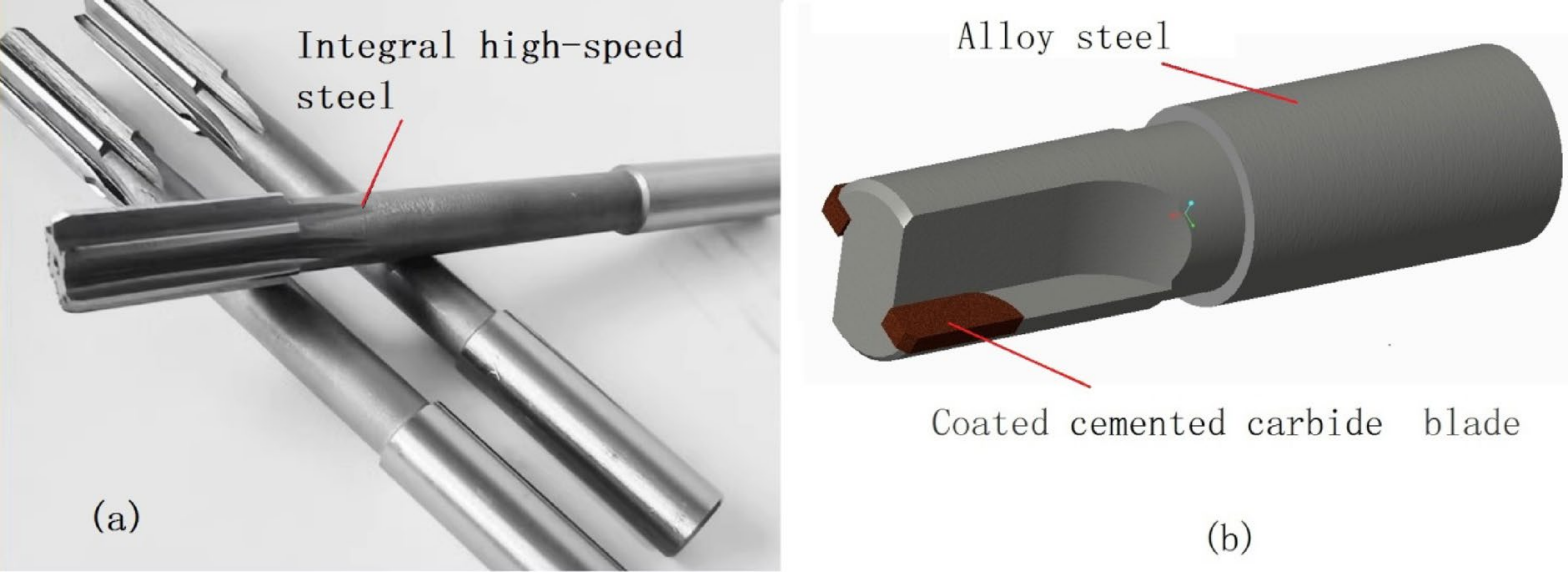
(**a**) Integral high-speed steel reamer (**b**) welded cemented carbide coated reamer.

A reamer is a complex and precise cylindrical cutting tool. The circumference of the reamer is equipped with multiple cutting teeth, and at the end of each tooth there is a small inclined cutting edge responsible for cutting the material of the workpiece. Due to its high machining accuracy and small material allowance (less than 0.2 mm), the cutting edges involved are very fine, which brings significant difficulties to the research. Ordinary reamers are generally made of high-speed steel, and their structure is shown in Fig. 1a. This type of reamer has low hardness, poor wear resistance, a low cutting speed (about 350 revolutions per minute), low machining efficiency, and is generally not used for cutting difficult-to-machine materials.

There have been numerous studies on the fabrication of cutting tools using TiN (TiCN, TiAlN, etc.) coatings deposited on the surface of cemented carbide materials through physical vapor deposition (PVD) or chemical vapor deposition (CVD) methods. Therefore, cemented carbide coated inserts are widely used in the manufacturing of cutting tools due to their low friction coefficient, high hardness, high wear resistance, and high efficiency^1–4^.

For the cutting of difficult-to-machine materials, there have been many studies on the manufacturing of turning tools^5–7^ and milling tools^8–12^ using coated cemented carbide inserts. The aim is to manufacture cemented carbide coated cutting tools with good performance under high cut-speed and high feed-rate machining conditions. Practice has shown that the application of cemented carbide coated inserts in turning tools and milling tools has been very successful. These experimental studies provide some references for high-efficiency precision machining with reamers. However, these experimental studies mainly focused on coated inserts with simple structures. They conducted machining experiments by welding or installing the inserts onto the tool body to manufacture the tools, and the analysis factors were relatively simple. Moreover, the influence on machining accuracy was rarely considered.

The cemented carbide coated reamer, due to its fine cutting edge and complex structure, and the closed machining environment, the influence of cutting heat, cutting force and wear on machining accuracy is comprehensive. The research is quite challenging. There are few studies on the reamer system aimed at improving the durability of the reamer. Only Ye et al.^13^ studied the chip-breaking principle of the cemented carbide coated reamer, but it adopted a welding structure, as shown in Fig. 1b. Zhang et al.^14^. conducted an experimental comparison of the cutting performance of two integral cemented carbide reamers with different helix angles, and only studied the effect of individual reamer parameters. These studies ignored the influence of high cutting-speed and high feed-rate machining conditions on the performance of the reamer, and had limited guidance for the design of the reamer system.

**Fig. 2:**
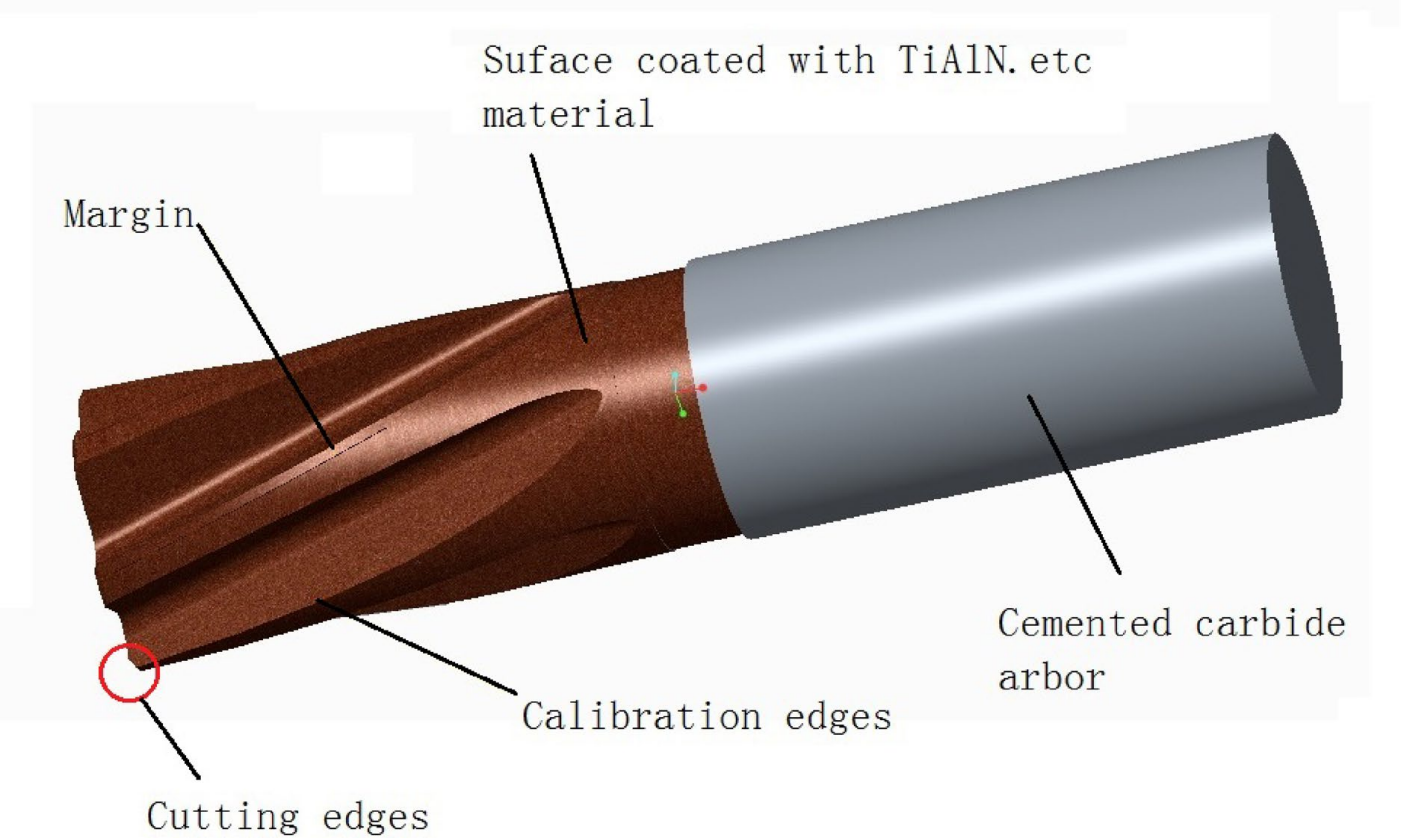
Integral cemented carbide coated reamer.

With the improvement of the comprehensive performance of cemented carbides^15–17^, it has become possible to manufacture integral cemented carbide reamers with complex shapes. However, under the high cutting-speed and high fast-rate machining conditions, the impact of cutting heat is significant, so internal cooling technology is needed to solve the problem. The application of internal cooling technology becomes extremely important. Xu et al.^18^ studied the internal cooling technology in cutting tools, analyzed the role of internal cooling technology in reducing cutting force and improving cooling effect during milling, and Farhana Diba et al.^19^, Guo et al.^20^ studied the implementation and analysis of internal cooling structures in drills. These technologies provide technical support for the cutting research of integral cemented carbide coated reamers. As shown in Fig. 2.

The research method we adopted is different from the experimental method. By setting a high rotational speed and a large feed rate to machine a certain difficult material, and ensuring the machining accuracy as the prerequisite, we comprehensively studied various factors that affect the machining capability of this type of tool. Only by adopting this relatively strict machining environment can the advantages of this high-performance tool be maximally exploited, and the obtained data will have true reference value.

## Analysis of the fracture of the cutting edge

###  Set processing conditions

Two positioning holes need to be precision machined on the 45 steel workpiece. The reaming tool will cut the hole with a diameter of Φ17.8 to Φ18. The precision requirements are an upper deviation of + 0.011, a lower deviation of -0.008, a hole depth of 15, and a surface roughness of Ra1.6. The machining process is carried out on a CNC machine. The reaming tool’s rotational speed is set at 3000 r/min, and the feed speed is 30 mm/s. Cooling and lubrication are achieved through external spraying.

### The initial design of the reamer

According to the cutting requirements, the cemented carbide round bar ETM210 produced by ETM Company was selected as the tool base. The parameters of this material are shown in Table 1. Firstly, an integral reamer was manufactured according to the parameters in Table 2, and then a TiAlN material was coated on it. The shape of this reamer is shown in Fig. 2.

**Table 1:** Main parameters of EMT 210 model Materials.

WC	Co	Average grain size(µm)	HRA	Transverse rupture strength(*N*/ mm²)
89%	10%	-0.8	92.1	> 4300

**Table 2:** Geometric information of reamer.

Reamer parameter	Value
Rake angle *γ*	5°
Clearance angle *α*	20°
Main cutting angle *Kr*	30°
Diameter of the cutting part *Dtool*	Φ18.001
Cutting layer thickness *ap*	0.1 mm
Number of cutting edges *Z*	6
Rake angle for circumference	3°
First Clearance angles for circumference	8°
Second Clearance angles for circumference	18°
Length overall	78 mm

###  The machining conditions and failure characteristics of the reamer

**Fig. 3:**
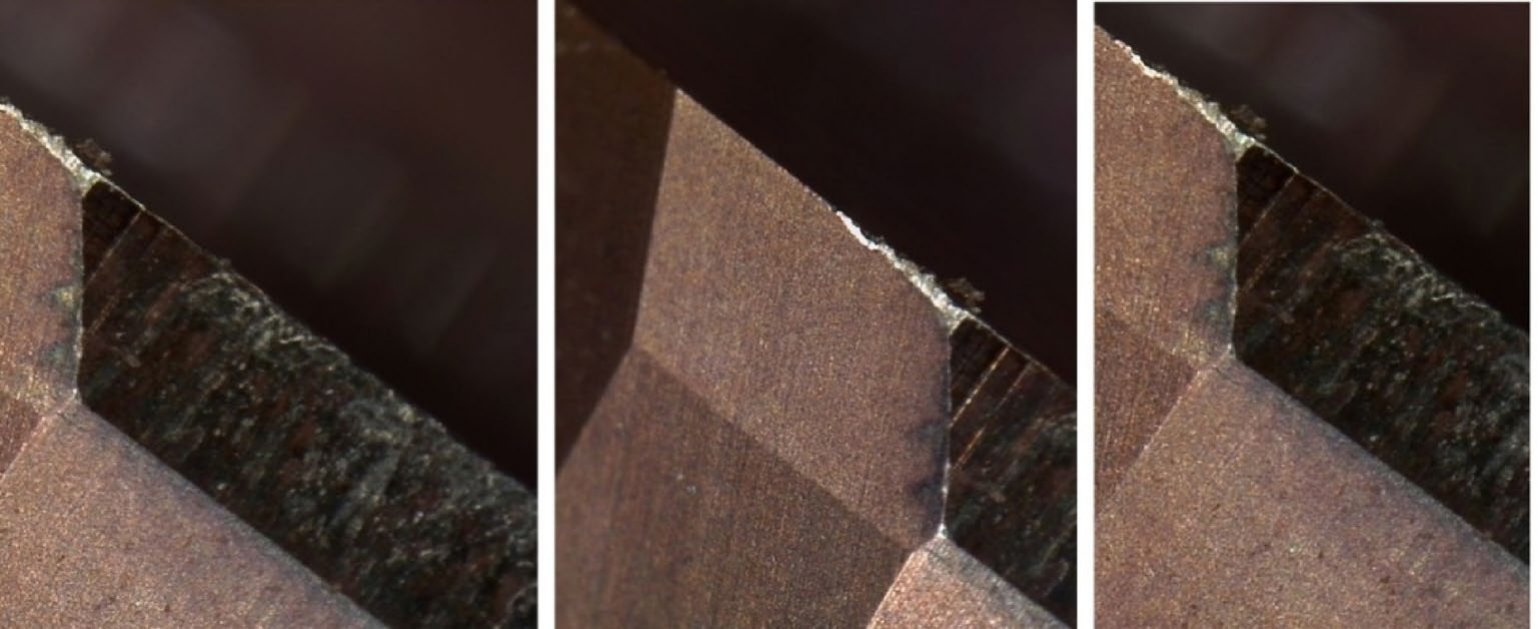
magnification of cutting edge failure.

During the testing process, after the reamer had machined 70 qualified workpiece holes, the 71st hole showed an error that exceeded the standard and failed to meet the machining accuracy, thus the tool became ineffective. It was difficult to find the reason for the failure of the micro-cutting edge visually. The micro-cutting edge of the reamer was photographed under an electron microscope, and the image of the failure of the micro-cutting edge is shown in Fig. 3. By observing the images of these three cutting edges, it was found that the cutting edges involved in the cutting had become wavy and rough, significantly different from the straight edges that did not participate in the cutting. This damage should be a fracture at the edge, with low cutting ability. It can be inferred that the reduction in the diameter of the workpiece hole was caused by the reduction in the material of the cutting edge.

### Changing the main cutting angle *Kr*

By analyzing the reasons for the breakage of the reamer, it is preliminarily concluded that the angle of the main cutting angle (*Kr*) is too small, which results in a shortened cutting edge and an excessively large load per unit length, causing the edge to break.

#### Theoretical advantages of changing the angle of the main cutting angle

**Fig. 4:**
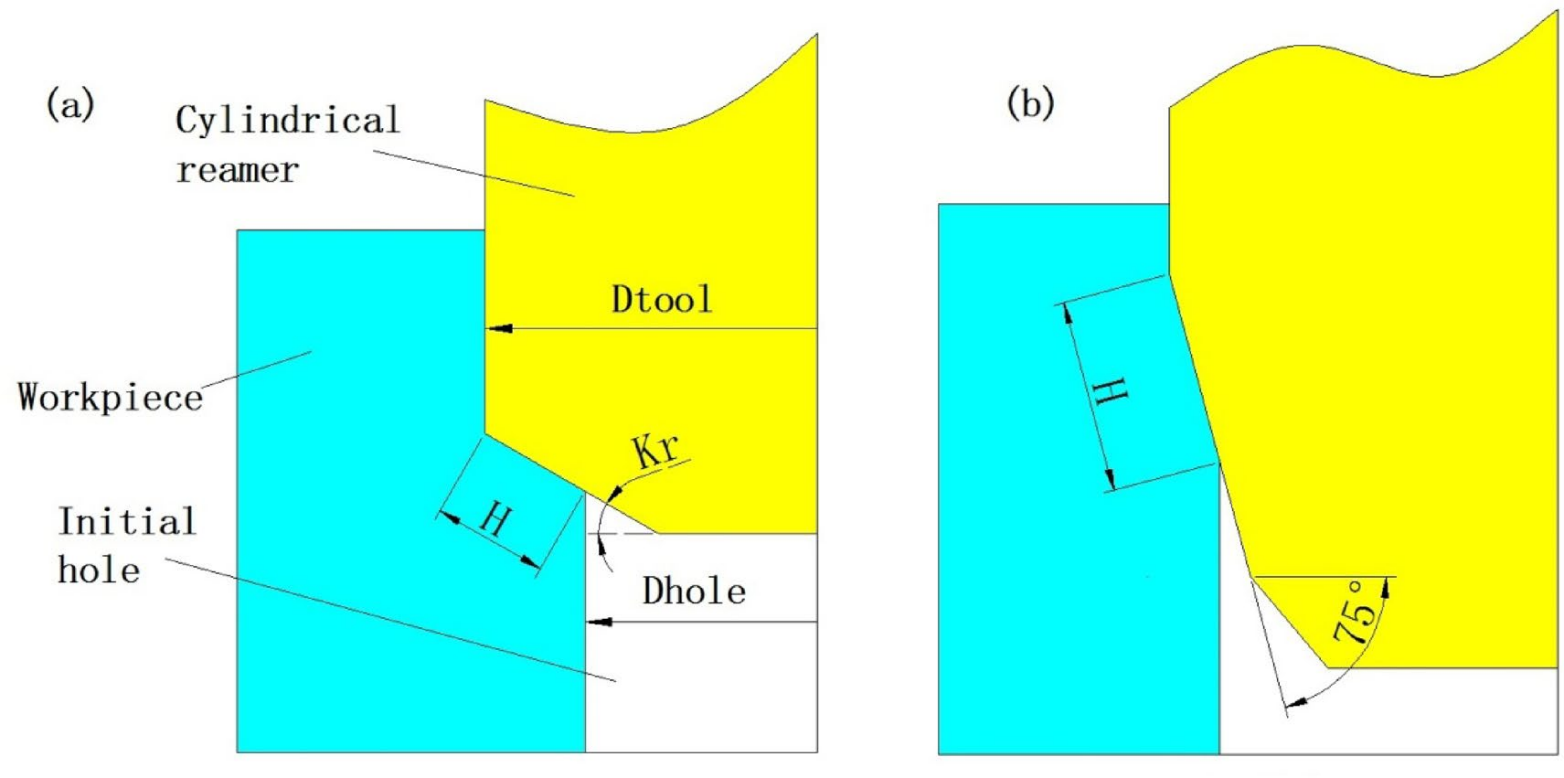
Machining schematic diagram of changing *Kr* (**a**) *Kr* = 30º (**b**) *Kr* = 75º.

To increase the length of the cutting edge involved in cutting the workpiece material, the angle of the main cutting angle (*Kr*) can be changed. The machining schematic diagram is shown in Fig. 4.

As can be seen from Fig. 4a, the length of the cutting edge involved in the cutting process is related to *H*, *Kr* and the cutting edge length *H* is expressed by Eq. (1).

1 $$\:H=\frac{Dtool-Dhole\:}{2\cdot \text{c}\text{o}\text{s}K\text{r}}$$

When *Kr* is changed to 75º, the cutting diagram of the tool is shown in Fig. 4b. The length of the cutting edge has significantly increased, approximately doubling.

#### The machining situation and failure characteristics of the 75º reamer

**Fig. 5:**
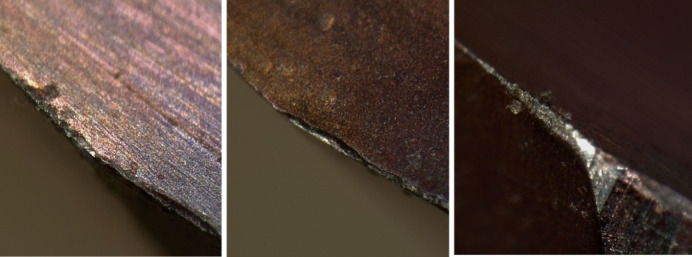
The enlarged view of the cutting edge failure when *Kr* = 75º.

When the reamer *Kr* = 75º and other parameters remain unchanged, after machining 150 qualified workpieces with this reamer, the minimum limit size of the hole exceeds the tolerance and the tool fails. The failure image of the cutting edge is shown in Fig. 5.

As can be seen from Fig. 5, there are gaps on the edge lines of the cutting edge or the calibration edge, and the material has broken off. The failure mode remains as a broken edge. Because more parts are being processed, the areas near the edge have darkened, which is a discoloration caused by high temperatures.

###  Analysis of broken edges

####  Mechanical model of the cutting edge

##### Derivation of the unilateral stress relationship

**Fig. 6:**
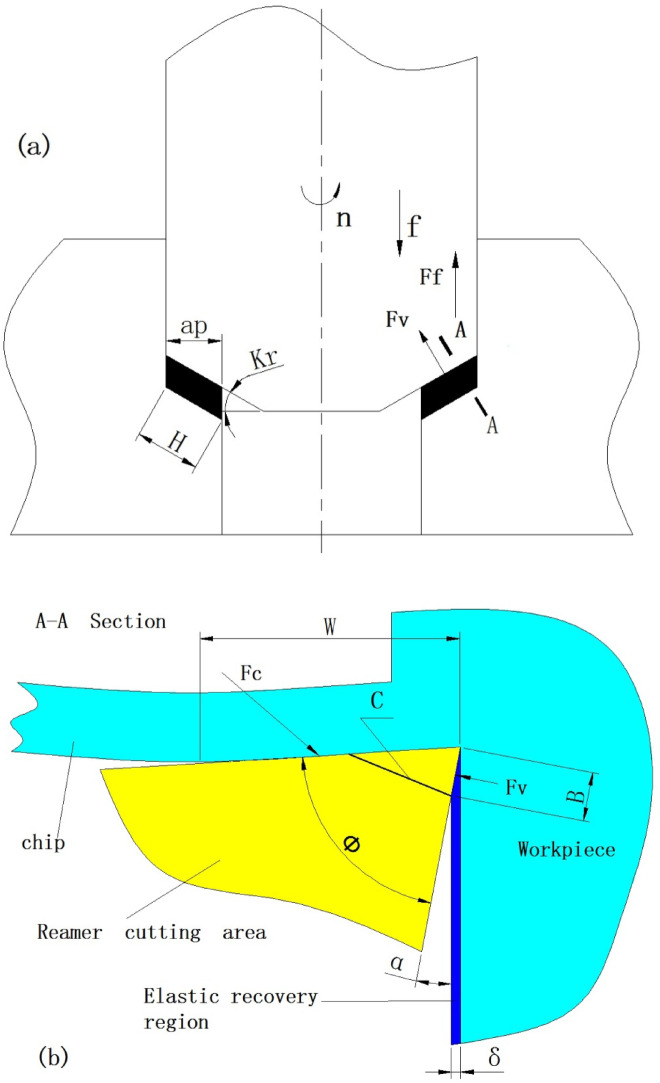
(**a**) Parameter diagram (**b**) stress diagram of cutting edge.

The parameters of the reamer cutting process are shown in Fig. 6a.

According to Fig. 6a, the reamer rotates at a speed of *n* and moves along the axial direction of the hole at a speed of *f*. The cutting edge area mainly bears the forces from the workpiece, including the main cutting force and the feed force. Among them, the main cutting force *Fc* is closely related to the reamer’s rotational speed *n*, feed speed *f*, and cutting layer depth *ap*, and can be expressed by the function f(*n*,* f*,* ap*), which is the total cutting force. Since there are *Z* cutting teeth involved in the cutting process, the main cutting force *Fc* on a single cutting edge can be represented by Eq. (2).

2 $$Fc=f(n,\:f,ap)/Z\:$$

Ref^13^ indicates that *Fc* is a relatively large constant at high rotational speeds, and its fluctuation is not significant as the feed rate f increases. The feed force *Ff* is mainly the reaction force of the workpiece along the direction opposite to the feed direction. Based on^13^, the calculation Eq. (3) for the feed force *Ff* can be derived.

3 $$\:Ff=\frac{Ks \cdot ap \cdot Z \cdot f}{{\uppi\:} \cdot D \cdot n}+\frac{Kf \cdot {\uppi\:} \cdot D \cdot n}{f}+C$$

Among them, *Ks* is the chip layer coefficient, *Kf* is the friction coefficient, *D* is the reamer diameter, and *C* is a constant. The force *Fv* perpendicular to the cutting edge is a component of *Ff*, which directly affects the fracture of the cutting edge and can be obtained by Eq. (4).

4 $$\:Fv=\frac{Ff \cdot \text{c}\text{t}\text{a}\text{n}Kr}{Z}$$

According to Eq. (4), if *Kr* increases, *Fv* will decrease.

##### The direction of force and the dangerous section

Figure 6b is an enlarged schematic diagram of section A-A in Fig. 6a, which shows the direction of the force and the area. The forward force *Fv* is mainly due to the elastic recovery of the surface of the machined hole. This elastic recovery is generated by the interaction between the elastic recovery material and the rear cutting surface of the tool. It is a kind of contact force, produced on the side, and the direction of the force should be perpendicular to the contact surface. The range of the force is the elastic contact area, and its size is represented by *B*, and can be calculated using Eq. (5).

5 $$B=\frac{\delta\:}{\text{t}\text{a}\text{n}a}$$

The elastic response *δ* is mainly determined by the workpiece material and the thickness of the chip layer *ap*. The contact area is mainly determined by the gap angle *α*.

The main cutting force *Fc* mainly consists of the deformation force of the chip and the friction force. Due to the coating on the surface of the tool material, the friction coefficient is small, and the proportion of the friction force is small. In other words, the component of *Fc* in the direction parallel to *Fv* is relatively large, and its effect range is mainly the area *W* where the rake face contacts the chip. This is mainly related to the material of the workpiece, and the influence of the tool’s rake angle is not significant.

Based on the positions, areas of action and blade structure characteristics of *Fc* and *Fv*, the section where the *C* position line is located is considered a dangerous section. The *C* position line is perpendicular to the lower end of the *B* line, so it is related to the size of the contact area *B*. The *C* line can be calculated using Eq. (5).

##### Mechanical model of the individual cutting edge of a single segment

**Fig. 7:**
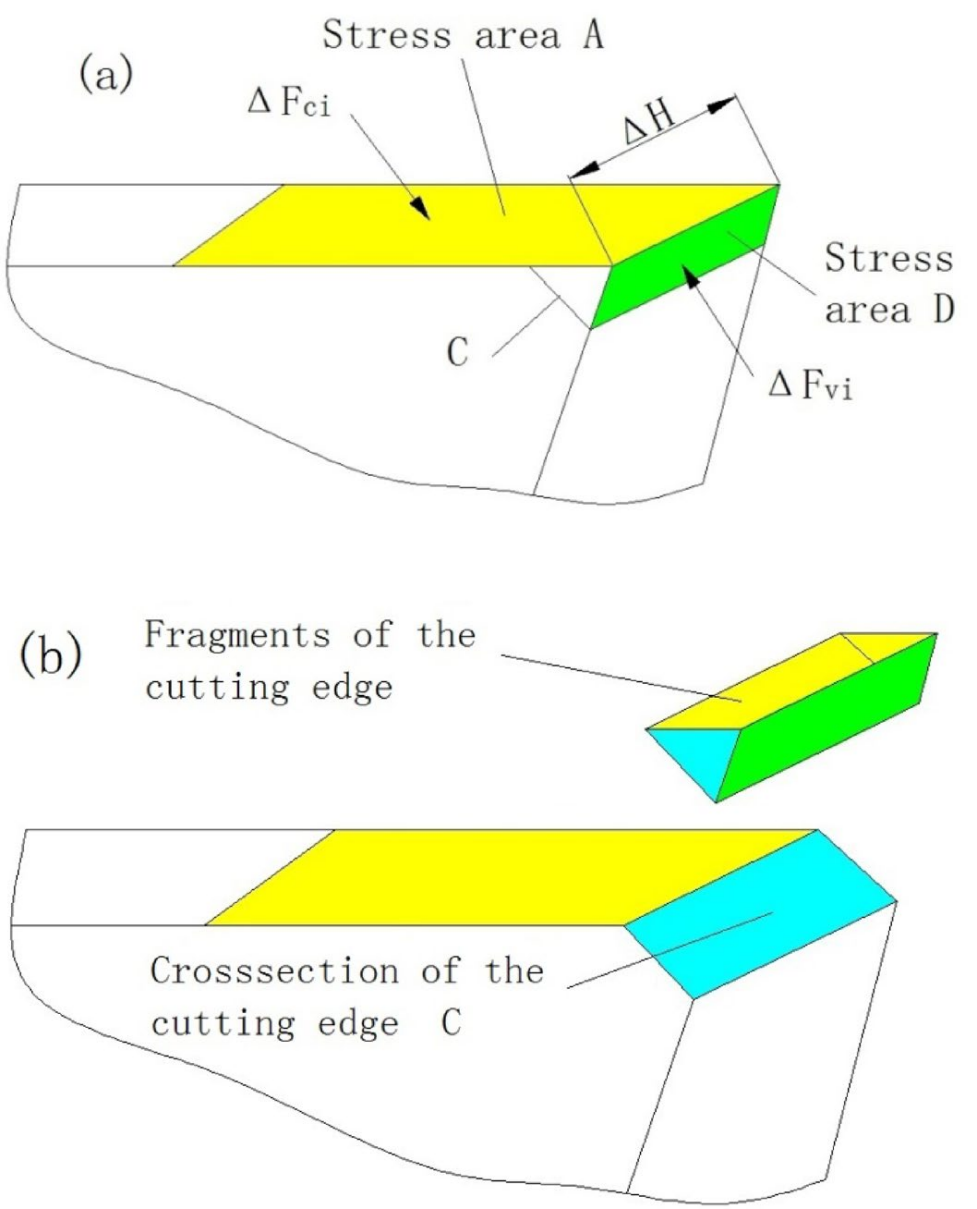
Schematic diagram of a single-stage micro-cutting edge. (**a**) Stress diagram (**b**) fracture diagram.

According to Fig. 6a, the length of the cutting edge is *H*. The cutting edge is divided into several small segments along the direction of the cutting edge, and a section with a length of *ΔH* is selected for analysis. Based on the cross-sectional Fig. 6b, the stress model of this section of the cutting edge is established, Fig. 7a. Since the cutting edge is composed of several *ΔH*, the main cutting force *Fc* can also be regarded as the sum of the cutting forces *ΔF*_*ci*_ of each section of the cutting edge, which is expressed by Eq. (6).

6 $$Fc=\sum\limits_{i=0}^{H}\left(\varDelta\:{F}_{ci}\right)$$

Assuming that the thrust force of each section cutting edge is *ΔF*_*vi*_, their relationship with the total thrust force *Fv* can be expressed by Eq. (7).

7 $$Fv=\sum\limits_{i=0}^{H}\left(\varDelta\:{F}_{vi}\right)$$

According to Eq. (6), the larger the *Fc* is, the greater the *ΔF*_*ci*_ will be; according to Eq. (7), if *Fv* increases, *ΔF*_*vi*_ will also increase. According to Fig. 7a and Fig. 6b, the range of *ΔF*_*ci*_ is the stress area *A*, with a length of *W*; the range of *ΔF*_*vi*_ is the stress area *D*, with a length of *B*, and their width is *ΔH.*

According to the mechanical model, the shear stress *ε* at the *C* position plane can be obtained as Eq. (8)

8 $$\varepsilon = \:\frac{{\varDelta\:F}_{vi}}{\varDelta\:H \cdot B \cdot \text{t}\text{a}\text{n}\varPhi\:}-\frac{1}{\varDelta\:H \cdot B \cdot \text{t}\text{a}\text{n}\varPhi\:} \cdot \frac{{\varDelta\:F}_{ci} \cdot B}{\text{W} \cdot \text{c}\text{o}\text{s}\varPhi\:}=\frac{\varDelta\:{F}_{vi \cdot } \cdot \text{t}\text{a}\text{n}\alpha\:}{\varDelta\:H \cdot \delta\: \cdot \text{t}\text{a}\text{n}\varPhi\:}-\frac{\varDelta\:{F}_{ci} \cdot \text{s}\text{i}\text{n}\varPhi\:}{\varDelta\:H \cdot W \cdot {\text{c}\text{o}\text{s}}^{2}\varPhi\:}$$

If the value of *ε* is larger, it is easier to break.

According to Eq. (8), when the feed component *ΔF*_*vi*_ increases or the Clearance angle *α* increases, the *ε* value will increase. For the cutting condition of high feed-rate, according to Eq. (3), (4), (7), *ΔF*_*vi*_ is of course larger; If the Clearance angle *α* increases again, according to Eq. (5), the length of line *B* will be reduced, the length of line *C* will also be reduced, and the shear stress area will be reduced, so that the *ε* value will be greatly increased.

On the contrary, if the Clearance angle *α* is reduced, it will increase *B*, or the included angle *Φ* between the rake face and the flank face will increase, which will make the *C* line longer and the *ε* value will decrease.

#### Analysis

##### Explanation of the process of cutting edge fracture

Assuming *ε*_*b*_ is the strength limit of the material, due to manufacturing defects, the *ε*_*b*_ values at different cross-sectional positions may vary. When the *ε* value at the *C* section on a certain *ΔH* on the cutting edge satisfies ε > ε_b_, the cutting tool material at the upper part of the *C* section on this part of the cutting edge will fracture, Fig. 7b, resulting in the first small-scale fracture and shortening of the cutting edge. However, the cutting continues. The shortening of the cutting edge causes an increase in the load on other parts, and the *ΔF*_*vi*_ on other *ΔH* segments increases, causing the shear stress *ε* at the *C* section to increase, and the brittle section material continues to fracture and detach. The cutting continues, and the cutting tool material fractures successively until the entire cutting edge of the cutting tool breaks, resulting in chipping and tool failure. This is the process of the cutting edge fracture and failure of the integral cemented carbide coated reamer. Based on the previous analysis, the fracture of the cutting edge occurs intermittently, and the length of each single *ΔH* segment for the fracture is different. Additionally, when some cutting edges fracture, the force conditions of other cutting edges will change, so the position of the *C* line for subsequent fractures will also change, thus resulting in the fracture shapes as shown in Figs. 3 and 5. It can be seen that the fracture morphology of the cutting edge conforms to this mechanical model.

##### The method of avoiding broken edges

Selecting a harder cemented carbide material with a larger *ε*_*b*_ value can reduce the probability of *ε* > *ε*_*b*_ occurring. Reducing the rake angle *γ* of the cutting tool to even be negative can increase the angle *φ*, allowing the *C* line to extend, which is beneficial for reducing the *ε* value. Reducing the relief angle *α* can increase the contact length *B* between the cutting tool’s flank face and the workpiece, thereby increasing the effective area of *ΔF*_*vi*_ and the included angle *φ*, which has a significant effect on reducing the *ε* value. Although increasing *Kr* helps to reduce *Fv* and *ΔF*_*vi*_, the reduction is not significant, and the number of machined workpieces slightly increases, but it does not change the result of the edge break. The design of the integral cemented carbide coated reamer can comprehensively consider the above factors.

## Analysis of cooling and lubrication effects

Change the rake angle of the reamer *γ* to 0°, the clearance angle *α* to 5°, *Kr* to 75°. Other machining conditions remain unchanged. Manufacture a integral cemented carbide coated reamer and conduct on-site machining again.

### External cooling machining

**Fig. 8:**
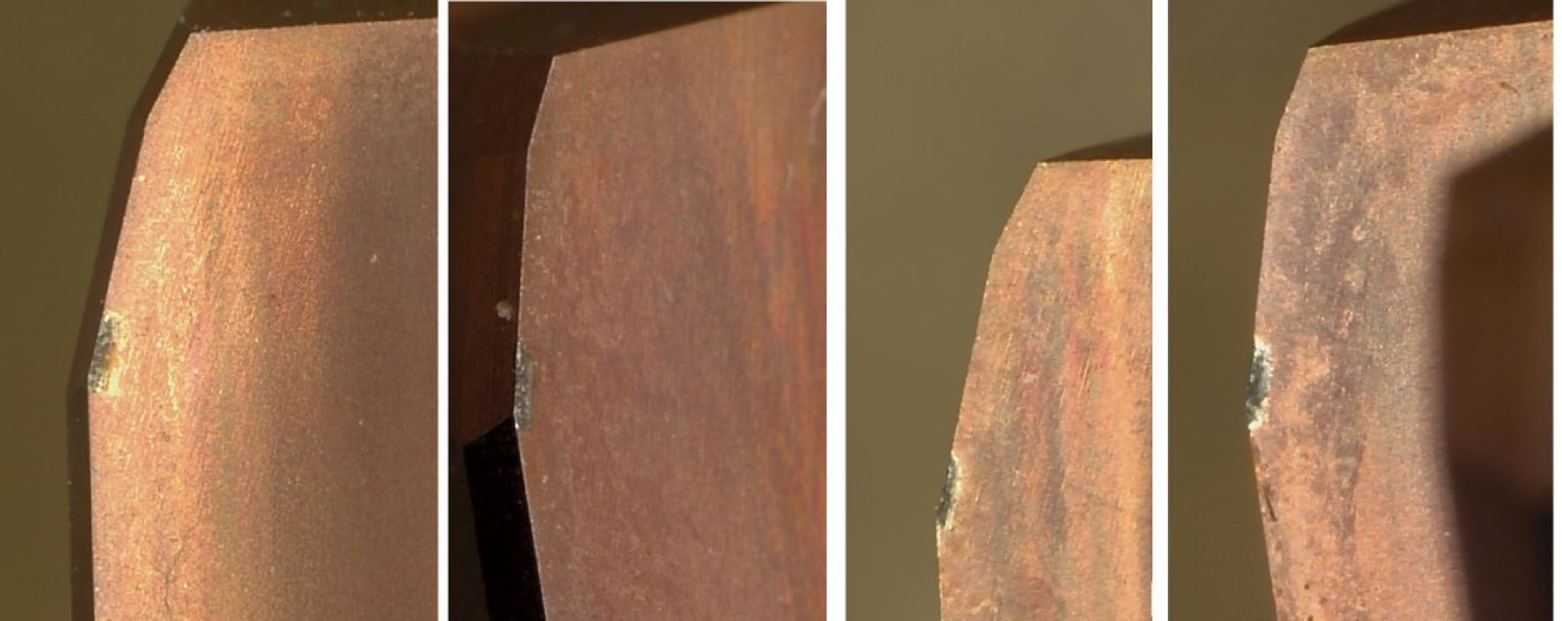
Failure diagram of cutting edge wear.

Using this reamer, 500 qualified workpieces were machined continuously. However, when it was used again, the size error of the holes exceeded the tolerance range, and the tool failed. By observing the micro-edge of the reamer under an electron microscope, the failure form of the micro-edge was obtained, as shown in Fig. 8.

Refer to Fig. 8. The reamer does not have a broken cutting edge. The failure mode is wear on the rake face. Moreover, part of the coating on the rake face has been worn away. The number of machined workpieces is not satisfactory. Such rapid wear failure should not occur^1–3^.

### Analysis of failure in machining with external cooling method

**Fig. 9:**
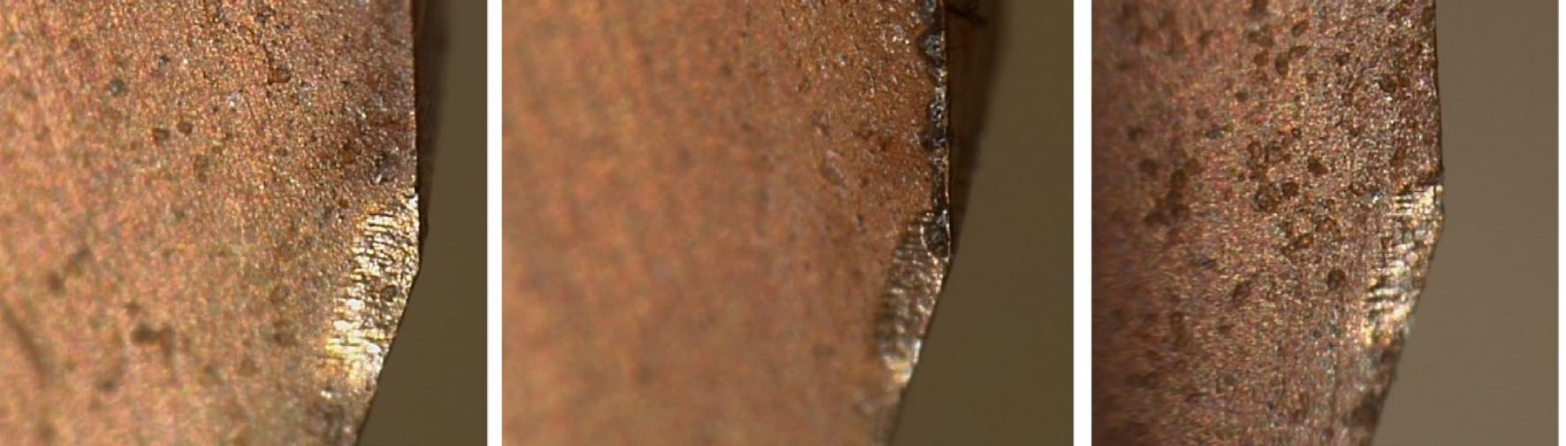
An enlarged image of the worn front cutting surface.

Further magnification of the failed cutting edge under the electron microscope was conducted to obtain a more detailed profile of the rake face wear, as shown in Fig. 9.

**Fig. 10:**
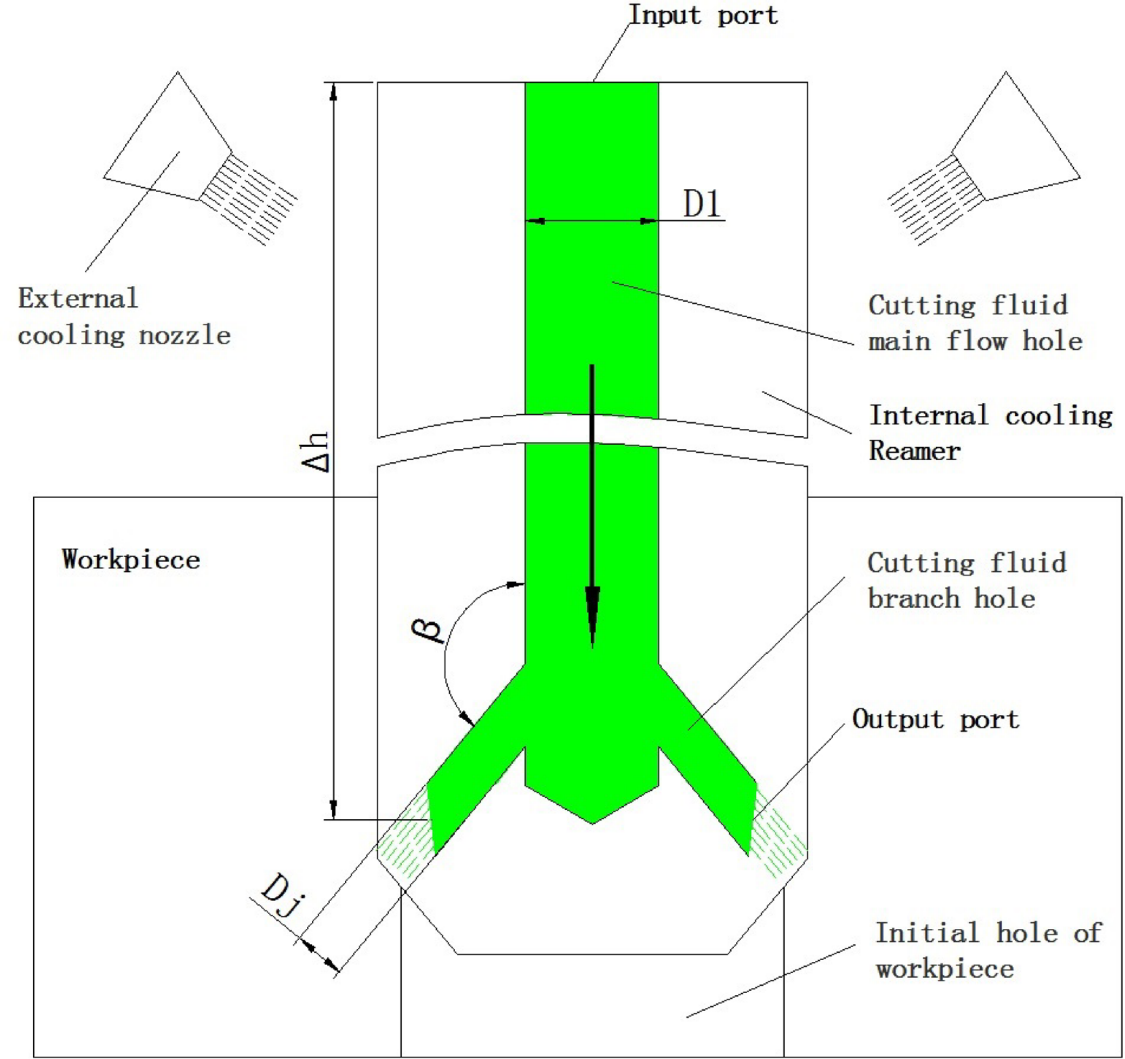
schematic diagram of cutting fluid cooling for Reamer.

It can be observed that the worn area on the rake face is not smooth and there are ablation spots. The worn area is not smooth, which should be caused by the built-up edges during the cutting process. The sequence, size and scraping depth of the built-up edges on the rake face are different. The presence of ablation spots indicates that the temperature in the processing area has risen too high, causing an oxidation reaction in the tool material. Both of these situations indicate that the cooling effect during the machining process is too poor, which is the reason for the rapid wear and failure of the reamer. The external cooling method involves the coolant flowing through the left and right nozzles to spray the cutting fluid onto the outside of the reamer, and then flowing into the hole to cool and lubricate the cutting edge, as shown in Fig. 10. However, due to the fact that the upper part of the reamer’s cutting edge is the alignment edge, pressing against the surface of the hole wall, and the tool rotating at high speed, it is difficult for the cutting fluid to flow into the cutting part of the cutting edge in time for cooling. The accumulation of cutting heat will cause the cutting area to rise in temperature too high, making the tool material become soft and oxidize, accelerating the wear. Therefore, this cooling method is ineffective.

### Research on internal cooling method

According to the research results in refs^18–21^, designing an internal cooling type reamer is beneficial for enhancing the cooling and lubrication effects and reducing the wear rate. We drilled a hole with a diameter of *D*_*1*_ at the center of the reamer, connected the cutting fluid to the center of the tool handle, and then the cutting fluid flowed out through a branch hole with a diameter of *D*_*j*_, spraying at a certain speed and pressure onto the cutting edge of the tool. Each tooth corresponds to a branch hole, and the angle between the main flow hole and the branch hole is *β*. The diameter and inclination angle *β* of each branch hole are the same. The structural form is shown in Fig. 10. To fully cool and lubricate the cutting edge, the cutting fluid at each branch outlet needs to have a certain speed *V*_*j*_ and pressure *P*_*j.*_ Therefore, the parameters *D*_*1*_, *D*_*j*_ and *β* need to be optimized. Usually, based on the parameters of the machine tool, the flow rate *Q*, speed *V*_*1*_ and pressure *P*_*1*_ of the cutting fluid flowing into the input port of the reamer can be calculated and determined. Equation (9) can be obtained based on the “Law of Conservation of Fluid Mass”.

9 $$Q = V_{1} \cdot A_{1} = \mathop \sum \limits_{{j = 0}}^{Z} V_{j} \cdot A_{j} = Z \cdot V_{j} \cdot A_{j}$$

Here: *Z* represents the number of branch holes, which is also equal to the number of teeth; *V*_*j*_ is the outlet speed of the branch; *A*_*1*_ is the cross-sectional area of the input port, which can be calculated using Eq. (10). *A*_*j*_ is the cross-sectional area of the output port, which can be calculated using Eq. (11).

10 $$A_{1} = \frac{{\pi D_{1}^{2} }}{4}$$

11 $$A_{j} = \frac{{\pi D_{j}^{2} }}{4}$$

Based on Eqs. (9), (10) and (11), the Eq. (12) for calculating *V*_*j*_ can be obtained.

12 $$\:{V}_{j}=\frac{{V}_{1} \cdot {D}_{1}^{2}}{{D}_{j}^{2}}$$

According to the “Bernoulli energy conservation law”, the liquid energy at the output port plus the loss is equal to the energy at the input port. The liquid energy relationship between the input port of the cutter’s main flow hole and each branch hole is obtained (Eq. 13).

13 $$\:\frac{{P}_{1}}{\rho\:g}+\frac{{V}_{1}^{2}}{2g}+\varDelta\:h=\frac{{P}_{j}}{\rho\:g}+\frac{{V}_{j}^{2}}{2g}+{h}_{1j}$$

Among them, *H*_*1j*_ represents energy loss, which is mainly related to *β*. The larger *β* is, the smaller *H*_*1j*_ will be. The appropriate range of *β* is 135º to 160º. *Δh* is approximately the tool length; *ρ* is the density of the cutting fluid; *g* is the gravitational acceleration. The Eq. (14) for calculating the output pressure *P*_*j*_ of the branch can be obtained from Eqs. (12) and (13).

14 $${P}_{j}={P}_{1}+\frac{{{\uprho\:}V}_{1}^{2}}{2}+(\varDelta\:h-{h}_{1j})\rho\:g-\frac{{\uprho\:}{V}_{j}^{2}}{2}$$

In practice, the values of parameters *D*_*1*_ and *D*_*j*_ fall within a certain range. The value of *D*_*j*_ should at least cover the cutting edge area. If *D*_*1*_ is large, the cutting fluid flow is sufficient, but it is prone to deformation when clamped, which affects the accuracy. After determining the structural parameters of the tool, combined with the parameters of the machine tool cooling system, Eqs. (12) and (14) can be used to check the flow velocity *V*_*j*_ and pressure *P*_*j*_ of the cutting fluid at the output port, which can help analyze the cooling effect.

## The performance and analysis of reasonable reamers

### Example of a reasonable reamer

**Fig. 11:**
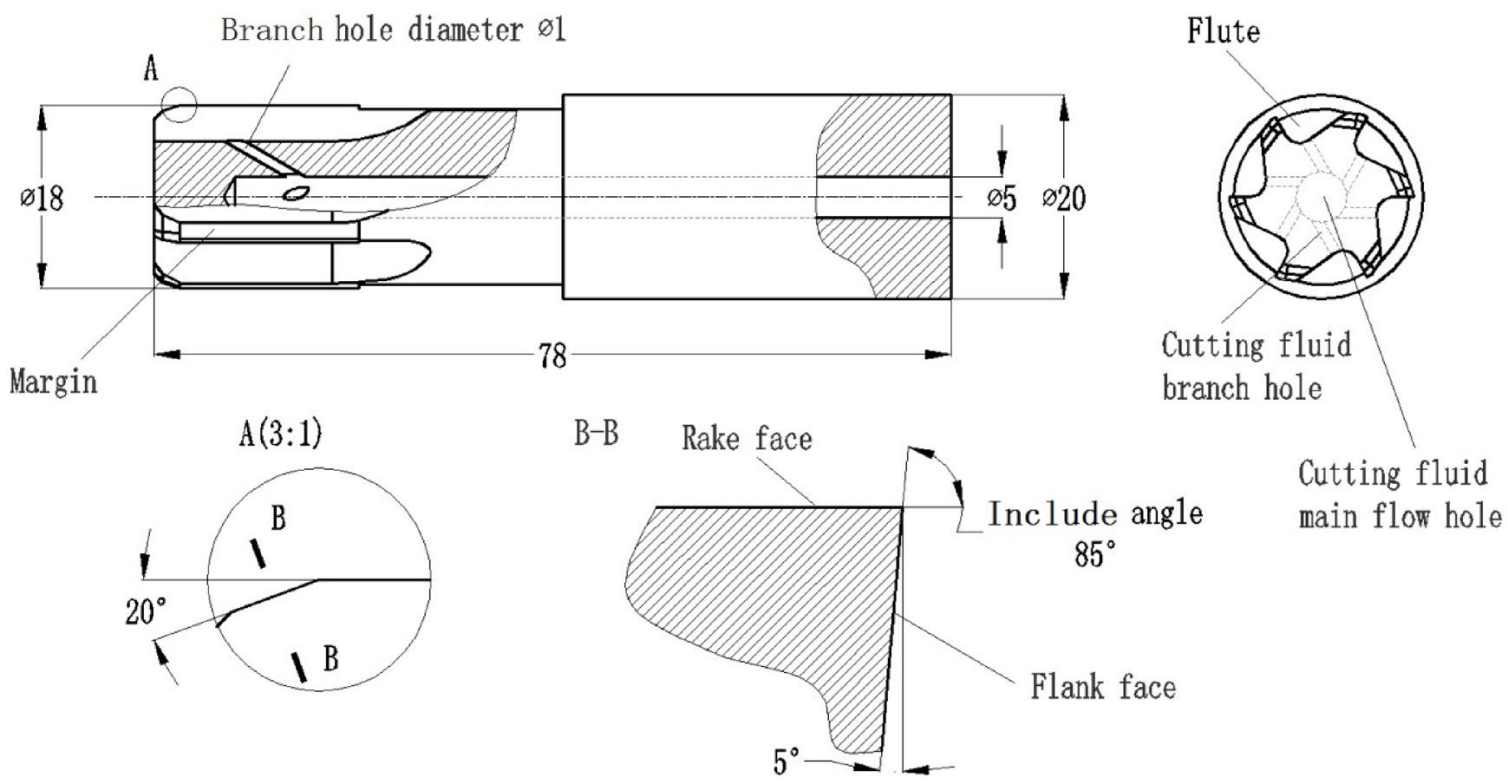
Optimized reamer sample illustration.

In order to ensure the machining accuracy, various factors analyzed in the previous section were comprehensively considered to design an integral cemented carbide coated reamer, and its machining was verified. The main structure and parameters are shown in Fig. 11; Table 3. The coated material is TiN^4^.

**Table 3:** Some geometric information of the reasonable reamer.

Reamer parameter	Value
Rake angle *γ*	0°
Number of cutting edges *Z*	6
Include angle *β*	150º
Margin width	0.5 mm

### Cutting performance

Using this sample reamer, more than 2000 qualified workpieces were continuously machined under high cutting-speed and high feed-rate conditions. This indicates that the wear of the tool has not exceeded the limit, the wear degree is small, and it proves that the tool wears slowly. The optimization results have significantly improved the durability and production efficiency of the tool, and fully demonstrated the superior cutting performance of this reamer, as well as verified the scientific nature of our research content.

### Analysis of wear conditions

**Fig. 12:**
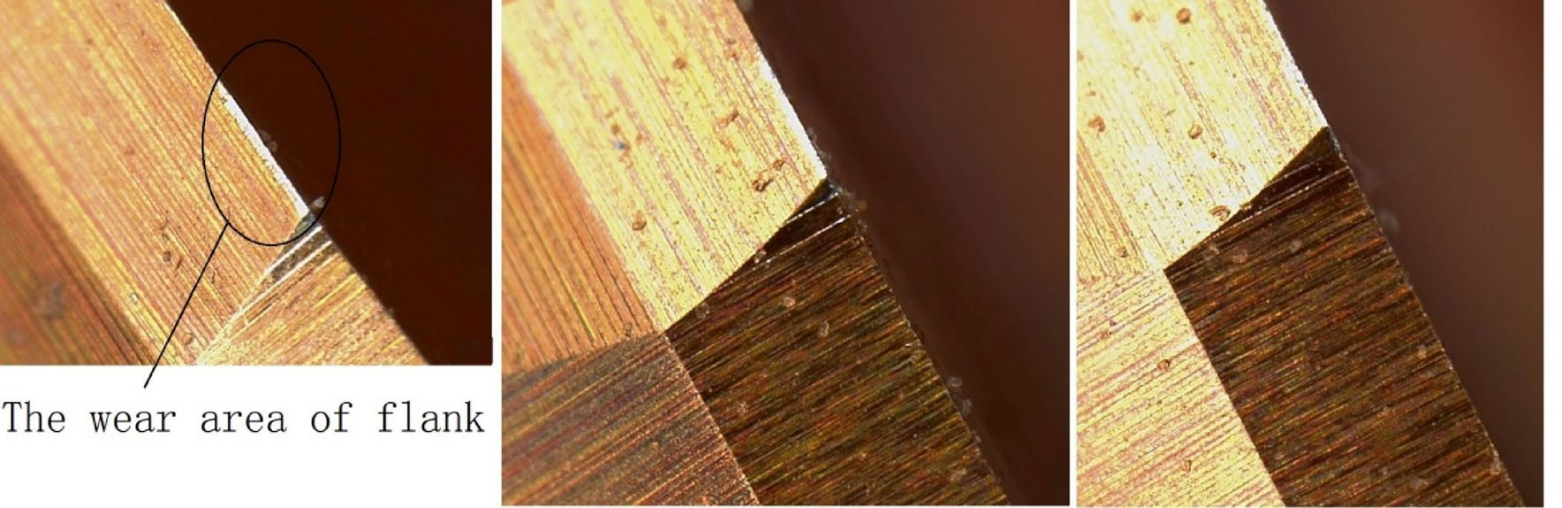
Wear appearance of flank face.

After machining 1200 workpieces with this reamer, an analysis and study were conducted on the wear of the cutting edges. Figure 12 shows the wear condition on the side at the cutting edge position. In the figure, the white band position represents the wear area of the flank face, with uniform wear, shallow wear marks, and similar wear on the flank face of each cutting edge, reflecting that the flank face had a large area of contact during the processing. Due to the good wear resistance of the material, the flank face was subjected to uniform force, so the wear was slow.

**Fig. 13:**
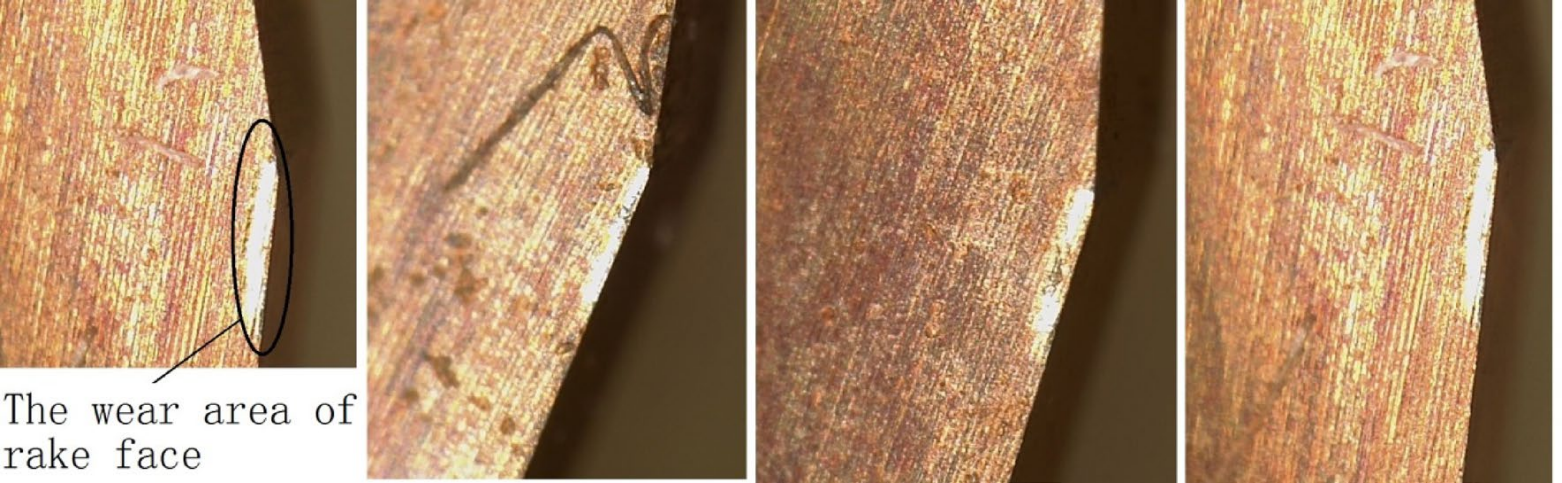
Wear appearance of rake face.

Figure 13 shows the wear condition of the rake face at the cutting edge position. The worn area is relatively wide, resembling a rectangle, and the wear is uniform with shallow scratches. This also reflects the uniform friction between the chip and the rake face, and the slow surface wear, which is closely related to the good wear resistance of the surface material and the good lubrication. Additionally, in the cutting areas of Figs. 12 and 13, there are no blackened or dimmed areas, indicating good cooling. This kind of graphic appearance is also an important reference to reflect the rationality of cutting tools.

From this, it can be seen that for this type of reamer that is machined under high cutting-speed, high feed-rate and in a closed environment, adequate lubrication and cooling for the cutting edge, as well as reasonable tool structure parameters, are important factors for maximizing its cutting performance.

## Conclusions

This integral cemented carbide coated reamer has the advantages of high efficiency, high precision, high wear resistance and high stability. It can also complete the precision hole machining of difficult-to-cut metals under conditions of high cutting-speed and feed-rate. To fully utilize the performance advantages of this reamer, several factors need to be comprehensively considered :

1. Although the cutting edge of the cemented carbide coated reamer is fine, it is still prone to rapid edge breakage during high feed-rate machining. Reducing the rake angle *α* (preferably less than 8°) or increasing the included angle *φ* between the front and rear cutting surfaces (preferably greater than 80°) is an important means to avoid edge breakage. The main cutting angle *Kr* should not be too small to avoid excessive concentration of cutting edge load and fracture.
2. When the reamer is externally cooled at high cutting- speed, the cutting fluid is difficult to reach the cutting edge, resulting in poor cooling and lubrication effect, and the cutting edge will accelerate wear. Good cooling and lubrication are important means to slow down wear failure.
3. In the internal cooling method, it is necessary to ensure that the cutting fluid has a certain pressure and speed when it is sprayed out from the branch holes, and can reach the cutting edge position.
4. A well-designed reamer must have an appropriate contact between the rake face and the chip, and between the flank face and the workpiece surface, so that the wear morphology of the front and rear cutting surfaces is regular and uniform, and the wear area is large, which can slow down tool wear and improve durability.

The magnified images of the wear and failure of the cutting edge of the reamer obtained in this scientific investigation are different from the magnified wear images of the cemented carbide coated inserts obtained under the experimental environment^1–12^. They are important reference materials for studying the micro-edge cutting of cemented carbide coated tools.

## Data Availability

The datasets used and/or analysed during the current study available from the corresponding author on reasonable request.
